# Fertility-sparing surgery of malignant transformation arising from mature cystic teratoma of the ovary

**DOI:** 10.18632/oncotarget.25548

**Published:** 2018-06-08

**Authors:** Nobuhisa Yoshikawa, Toshiya Teshigawara, Yoshiki Ikeda, Kimihiro Nishino, Jun Sakata, Fumi Utsumi, Kaoru Niimi, Ryuichiro Sekiya, Shiro Suzuki, Michiyasu Kawai, Kiyosumi Shibata, Fumitaka Kikkawa, Hiroaki Kajiyama

**Affiliations:** ^1^ Department of Obstetrics and Gynecology, Nagoya University Graduate School of Medicine, Nagoya, Japan; ^2^ Department of Obstetrics and Gynecology, Fujita Health University Banbuntane Hotokukai Hospital, Nagoya, Japan; ^3^ Department of Obstetrics and Gynecology, Toyohashi Municipal Hospital, Toyohashi, Japan

**Keywords:** malignant transformation, mature cystic teratoma, fertility-sparing surgery, oncological outcome, reproductive outcome

## Abstract

**Background:**

The purpose of this study was to evaluate the long-term clinical outcome of young women with malignant transformation arising from mature cystic teratoma of the ovary (MT-MCT) by comparing radical surgery and fertility-sparing surgery (FSS).

**Patients and methods:**

All patients treated with radical surgery or FSS for MT-MCT in multiple institutions were registered in this analysis. Univariate and multivariate analyses were performed to evaluate clinical outcome, including overall survival (OS) and disease-free survival (DFS).

**Results:**

From 1986 to 2016, 62 patients with MT-MCT were treated in our group. The median follow-up period was 38.0 (2.0-227.9) months, and the median age was 54 (17-82) years old. Multivariate analysis revealed that only advanced stage was significantly correlated with poorer prognosis of patients [hazard ratio (HR) for death: 6.58, 95% confidence interval (CI): 1.82–24.78, P = 0.0048; HR for recurrence: 5.59, 95% CI: 1.52–21.83, P = 0.01]. Of a total of 13 women with stage I-II disease at less than 45 years old, 7 were treated with FSS, and there was no recurrence except for in one woman with stage II MT-MCT. There was no significant difference in long-term oncological outcome between radical surgery and FSS.

**Conclusion:**

FSS may be indicated for patients with stage I MT-MCT, who hope to preserve fertility, as no relapse was found after FSS.

## INTRODUCTION

Usually, fertility-sparing surgery (FSS) has been acceptably chosen for young patients with an ovarian-confined / capsulated / well-differentiated epithelial ovarian cancer (EOC), as well as those with borderline, germ cell, and stromal tumor. Previous studies reported FSS as an alternative therapy to radical surgery in selected patients of child-bearing age, when the oncological outcome of patients treated with FSS was determined to be equivalent to that of patients treated with radical surgery [1–6]. However, evidence supporting the safety and effectiveness of conservative surgery is limited, and a standard indication for FSS remains controversial even for EOC, because randomized clinical trials are difficult to conduct due to ethical issues.

Mature cystic teratoma (MCT) is a common asymptomatic disease in women of child-bearing age, comprising up to a quarter of all ovarian tumors, with an incidence of 1.2-14.2 cases per 100000 per year [7–10]. MCT normally contains mature tissues from all three germ-cell layers (ectoderm, mesoderm and endoderm). It has been reported that malignant transformation of MCT (MT-MCT) usually occurs only in approximately 1-3% of MCT cases [9]. The most common malignant histological type of MT-MCT is squamous cell carcinoma (SCC) originating from ectodermal tissue, which accounts for 80-90%, but adenocarcinomas, carcinoid tumors and melanomas have also been reported infrequently [9, 11–13]. Although MT-MCT is normally diagnosed at menopausal age, our colleagues reported at least one fourth of MT-MCT actually occurs in reproductive-age women [14]. Due to its rarity, MT-MCT is not even independently categorized as a type of histology into the National Comprehensive Cancer Network Guidelines 2017. To our best knowledge, there has been no study assessing the applicability of FSS for MT-MCT.

This study was aimed to overview the oncologic outcome patients in our experience and explore the eligibility of FSS for MT-MCT. An insight into the valid management strategies for MT-MCT in child-bearing age was proposed.

## RESULTS

From 1986 to 2016, a total of 62 patients with MT-MCT were registered to this study. The clinicopathological characteristics of these patients are summarized in Table 1. The median follow-up time and the median age of patients were 38.0 (2.0-227.9) months and 54 (17-82) years old. Of a total of 62 patients, 38 patients had stage I disease, 5 had stage II disease, 17 had stage III, and 2 had stage IV, respectively. A total of 17 (27.4%) patients were treated with conservative surgery instead of radical surgery. Squamous cell carcinoma accounted for more than 90% of our cases. We first overviewed oncologic outcome of patients in our experience. Kaplan-Meier curves of OS and DFS, demonstrating demonstrated both 5-year OS and DFS were 64%. In order to identify possible prognostic factors affecting OS and DFS, univariate and multivariate analyses were performed (Table 2). Age, surgical procedures, residual tumor at initial surgery, adjuvant chemotherapy, SCC and CA125 did not influence OS or DFS in both univariate and multivariate analyses. Only FIGO stage was a significant prognostic factor for both OS and DFS in multivariate analyses (OS: HR: 6.58, 95% CI: 1.82-24.78, P =0.0048; DFS: HR: 5.59, 95% CI: 1.52-21.83, P =0.01). In fact, 5-year OS was as follows: stage I-II, 78%; stage III-IV, 32%. The prognosis of patients with early stage was much more favorable than in those with advanced stage, therefore FSS may be acceptable for patients with early stage MT-MCT who hope to preserve fertility.

**Table 1:** Patient characteristics

	n (%)
Age, years	
median age (range)	54 (17-82)
≧60	25 (40.3)
45-59	17 (27.4)
<45	20 (32.3)
Stage	
I	38 (61.3)
IA	22
IC	16
II	5 (8.1)
IIA	1
IIB	2
IIC	2
III	17 (27.4)
IIIB	7
IIIC	10
IV	2 (3.2)
Surgical Procedures	
Radical surgery	45 (72.6)
Complete surgery^*^	15
Incomplete surgery^**^	30
Conservative surgery	17 (27.4)
Residual tumor	
Yes	53 (85.5)
No	9(14.5)
Histology	
Squamous cell carcinoma	57 (91.9)
Adenocarcinoma	3 (4.8)
Adenosquamous carcinoma	1 (1.6)
Malignant melanoma	1 (1.6)
SCC (ng/dL)	
< 2	9 (14.5)
≧ 2	31 (50.0)
Unknown	22 (35.5)
CA125 (U/mL)	
< 100	45(72.6)
≧ 100	15 (24.2)
Unknown	2 (3.2)
Adjuvant Chemotherapy	
Yes	37 (59.7)
No	19 (30.6)
Unknown	6 (9.7)

Complete surgery^*^ was defined as completion of all procedures in radical operation including total hysterectomy and bilateral salpingo-oophorectomy with peritoneal staging and lymphadenectomy. Incomplete surgery^**^ was defined as completion of total hysterectomy and bilateral salpingo-oophorectomy.

**Table 2:** Uni- and multivariate analyses of clinicopathologic parameters in relation to OS and DFS of patients

Variable	Univariate			Multivariate		
	HR	95%CI	*P*-value	HR	95%CI	*P*-value
Overall survival						
Age (≧45 versus <45)	0.68	0.28-1.75	0.41	0.53	0.19-1.49	0.22
Stage (III-IV versus I-II)	6.14	2.50-15.9	<0.0001	6.58	1.82-24.78	0.0048
Surgical procedures (Conservative versus Radical)	0.41	0.10-1.24	0.12	0.75	0.13-3.30	0.71
Residual tumor (Yes versus No)	3.74	1.32-9.36	0.02	0.76	0.21-2.74	0.67
SCC (≧2.0 versus <2.0)(ng/dL)	2.20	0.59-14.27	0.26	1.30	0.25-10.50	0.77
≧100 versus <100)(U/mL)	1.57	0.59-3.84	0.35	1.25	0.44-3.33	0.66
Chemotherapy (Yes versus No)	205	0.73-7.24	0.18	0.79	0.23-3.13	0.72
Disease-free survival						
Age (≧45 versus <45)	0.69	0.29-1.78	0.43	0.57	0.20-1.63	0.29
Stage (III-IV versus I-II)	5.43	2.22-14.01	0.0002	5.59	1.52-21.83	0.01
Surgical procedures (Conservative versus Radical)	0.43	0.10-1.30	0.14	0.86	0.16-3.75	0.84
Residual tumor (Yes versus No)	3.50	1.23-8.76	0.02	0.81	0.23-2.93	0.75
SCC (≧2.0 versus <2.0)(ng/dL)	1.97	0.53-12.73	0.34	0.99	0.20-7.41	0.99
≧100 versus <100)(U/mL)	1.69	0.63-4.13	0.28	1.27	0.45-3.30	0.64
Chemotherapy (Yes versus No)	2.09	0.32-12.03	0.36	0.86	0.25-3.42	0.82

**Figure 1:**
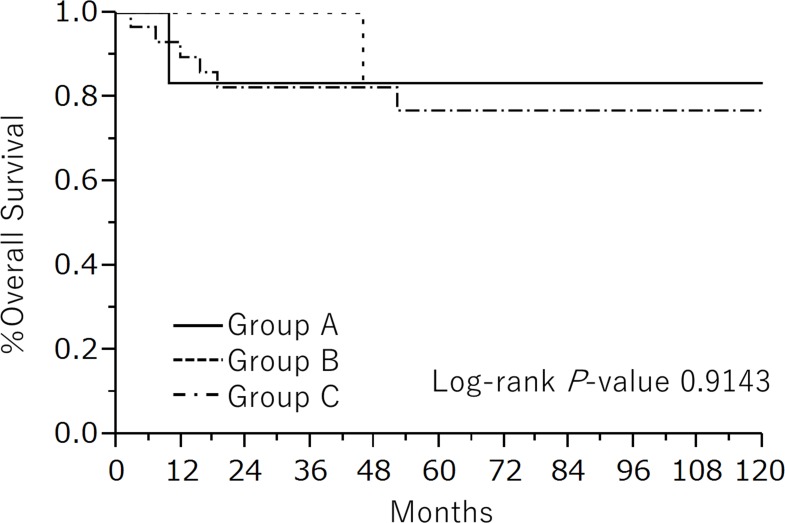
Kaplan-Meier estimated OS of early stage MT-MCT patients divided into three groups: group A, patients who underwent FSS under 45 years old; group B, patients who underwent radical surgery under 45 years old; group C, patients over 46 years old

We subsequently explore the possibility of FSS in young patients with MT-MCT. The clinicopathological features of patients with early stage disease among group A, B, and C are summarized in Table 3. Of seven patients in group A, five patients underwent unilateral salpingo-oophorectomy, and the other two patients received unilateral cystectomy. We selected FSS for the patient with stage II MT-MCT because they wished to preserve fertility regardless of our recommendation for radical surgery, which resulted in recurrence. Three patients treated with FSS underwent adjuvant chemotherapy. The clinical characteristics excluding surgical procedures were not statistically different among the three groups. Figure 1 shows the Kaplan Meier curves for OS among three groups, and 5-year OS in the individual groups was as follows: group A, 83%; group B, 83%; and group C, 77%. No significant difference regarding long-term survival was found among these groups.

**Table 3:** Clinicopathological features of early stage MT-MCT among group A, B, and C

	Group A (n=7)	Group B (n=6)	Group C (n=30)	*P*-value
Stage				0.2965
I	6	5	27	
IA	4	2	16	
IC	2	3	11	
II	1	1	3	
IIA	1	0	0	
IIB	0	0	2	
IIC	0	1	1	
Surgical procedures				0.0032
Radical surgery	0	6	21	
Complete surgery	0	4	7	
Incomplete surgery	0	2	14	
Conservative surgery	7	0	9	
Histology				0.8416
Squamous cell carcinoma	6	6	28	
Adenocarcinoma	0	0	1	
Adenosquamous carcinoma	1	0	1	
Malignant melanoma	0	0	0	
SCC (ng/dL)				0.8818
< 2	2	1	5	
≧ 2	2	3	15	
Unknown	3	2	10	
CA125 (U/mL)				0.35
< 100	6	5	21	
≧ 100	1	0	8	
Unknown	0	1	1	
Adjuvant Chemotherapy				0.8475
Yes	3	4	16	
No	3	2	12	
Unknown	1	0	2	

With regard to obstetrical outcome after FSS, two patients gave birth to healthy children. One patient became pregnant and gave birth to a healthy child after FSS, and the other patient underwent unilateral cystectomy for MT-MCT at 16 weeks pregnant, and then had a baby by cesarean section.

All 25 MT-MCT patients treated with conservative surgery listed in Table 4 were derived from previous representative reports and our current study [15–24]. The median age of these patients was 31 years old (19-41). Only one patient with stage II disease in our study relapsed (3.8%) and died due to disease progression. Except for this patient, the other 25 patients had stage I disease. Eight out of 25 patients (32%) with stage I MT-MCT received adjuvant chemotherapy following FSS, including paclitaxel and cisplatin. With respect to surgical procedure, unilateral oophorectomy with or without omentectomy and lymphadenectomy were mainly performed as FSS.

**Table 4:** Summary of clinical outcomes and fertility results of MT-MCT patients treated conservatively in our current study and previous reports

Case ID	Author	Age	Stage	Primary Surgery	Histology	Adjuvant Chemotherapy	Chemotherapy (course)	Status	Overall Survival (months)	Pregnancy outcome
1	Current report	41	IA	RSO	SCC	No		NED	226	-
2		30	IA	RSO	SCC	No		NED	131	1 (NVD)
3		33	IA	RSO + App	SCC	Yes	PTX + CBDCA (3)	NED	60	-
4		40	IC	RSO	SCC	No		NED	24	-
5		38	IA	left cystectomy	SCC	Yes	PTX + CBDCA (3)	NED	10	-
6		31	IIA	RSO + left cystectomy + OM	SCC	Yes	CDDP + 5FU (1) GEM + CBDCA (4) + Bev (3)	DOD	10	-
7		30	IC	right cystectomy	AC	NA		NED	11	1 (CS)
8	Tseng	31	IA	USO	SCC	-	-	NED	51	NA
9		21	IA	USO	SCC	-	-	NED	105	NA
10		22	IA	LSO	SCC	-	-	NED	121	NA
11		34	IA	USO	SCC	-	-	NED	91	NA
12	Rim	19	IA	LSO	SCC	-	-	NED	5	NA
13		36	IA	LSO	SCC	-	-	NED	20	NA
14	Arioz	31	IIA	USO + OM + PLND + PALND + App	SCC	Yes	PTX + CDDP (6)	NED	6	NA
15	Gainford	31	IA	USO	SCC	Yes	CDDP + ETP (5)	NED	108	NA
16		21	IA	USO	SCC	-	-	NED	12	NA
17		27	IA	USO	SCC	-	-	NED	48	NA
18		28	IC	USO + PALND	SCC	Yes	CDDP + ETP (3)	NED	18	NA
19	Sakuma	36	IA	LSO	SCC	-	-	NED	72	NA
20	Budiman	41	IA	RSO	SCC	-	-	NED	37	1 (NVD)
21	Chiang	32	IA	RSO	SCC	Yes	BEP (6)	NED	104	NA
22	Yun	30	-	LSO + right cystectomy	SCC	-	-	NED	60	1 (NVD)
23	Koc	28	IC	LSO	SCC	Yes	PTX + CDDP (3)	NED	96	NA
24		35	IA	LSO	SCC	-	-	NED	114	NA
25		38	IA	LSO + PLND + PALND + OM + App	SCC	-	-	NED	96	NA

Abbreviations: RSO: right salpingo-oophorectomy, App: appendectomy, USO: unilateral salpingo-oophorectomy, LSO: left salpingo-oophorectomy, OM: omentectomy, PLND: pelvic lymph node dissection, PALND: para-aortic lymph node dissection, SCC: squamous cell carcinoma, AC: adenocarcinoma, NA: not available, NED: no evidence of disease, DOD: died of disease, NVD: normal vaginal delivery, CS: cesarean section.

## DISCUSSION

Preservation of reproductive function is strongly desired by patients with ovarian malignancy at child-bearing age. Previous reports analyzing the clinical outcomes of women who received the surgical procedure have revealed the possibility of preserving fertility in early stage EOC [2, 25]. Regarding MT-MCT, there is currently no study evaluating eligibility for FSS or proposing criteria for FSS. Our current study is the largest case series study to evaluate prognostic factors affecting long-term clinical outcome of patients with MT-MCT, and to propose recommended criteria for FSS for MT-MCT.

Preoperative diagnosis of MT-MCT is difficult, especially in early stages, thus MT-MCT is usually diagnosed by postoperative histopathological examination. Moreover, like other ovarian malignancies, it is reported that the prognosis of this disease depends on the stage at diagnosis [9]. A previous study on 37 patients with MT-MCT reported that the 5-year survival rates was 94.7% for stage I, 80.0% for stage II and 0% for stage III and stage IV, indicating that patients with the disease in FIGO stage I have a relatively good prognosis [26]. Indeed, the 5-year OS of our patients with early stage MT-MCT was more favorable than that of patients with advanced stage MT-MCT in the total population. The FIGO stage at diagnosis was the only determinant of survival outcome according to our multivariate analysis. We suggest that conservative surgery may acceptable only for patients with early stage disease, and should not be applied to patients with advanced stage, although it is difficult to correlate the prognosis of these patients with surgical procedures.

Compared with previous reports, 5-year OS and DFS of stage I MT-MCT in this study were likely to be equivalent with those of stage I EOC [3, 9, 26, 27]. On the other hand, the prognosis of stage II MT-MCT was poorer than that of stage I disease, as in previous stage II MT-MCT.

Optimal surgical procedures for MT-MCT are undefined, compared with those for EOC. According to the National Comprehensive Cancer Network Guidelines 2017, FSS including USO preserving the uterus and contralateral ovary may be considered for patients with apparent early-stage disease and/or good-risk tumors, including malignant germ cell tumors, who hope to preserve fertility, whereas complete staging surgery is recommended for patients not desiring fertility preservation [28]. However, there are no criteria regarding FSS for MT-MCT. To our best knowledge, there are only a few reports regarding FSS for patients of reproductive age with MT-MCT who desire future fertility. Gainford et al. reported that FSS may be a reasonable choice in selected patients with stage I disease, based on their experiences [18]. However, we think that the number of patients are too small-scale to draw a reliable conclusion with respect to validity of FSS. In this study, we presented the clinical outcomes of a total of 62 patients with MT-MCT, and our results demonstrated no correlation between prognosis and surgical procedure by both univariate and multivariate analyses. Furthermore, in our study, not only unilateral salpingo-oophorectomy but unilateral cystectomy was performed as FSS for stage I disease, with no relapse. MT-MCT is frequently diagnosed at postoperative histopathological examination even if the preoperative diagnosis was benign. Our results suggest that patients treated with unilateral cystectomy may not need to undergo additional radical surgery following primary surgery.

According to the comparison of the younger age group (≤45) with the older age group (>45) in the univariate analysis, the survival differences in OS and DFS were not significant. Additionally, in the younger age group, 5-year OS and DFS of patients undergoing FSS were likely to be equivalent with those of patients who underwent radical surgery. Limited to stage I disease, there was no recurrence in both patients treated with FSS and in patients treated with radical surgery. However, we observed recurrence following FSS in the patient with stage II MT-MCT due to her hope to preserve fertility. Our results suggest that FSS may be an acceptable choice for patients with stage I MT-MCT but not for those with stage II disease.

As summarized in Table 4, there was no relapse in a total of 25 patients with stage I disease treated with FSS, therefore, conservative surgery for patients with MT-MCT may be applicable at least for stage I MT-MCT. Although the significance of adjuvant chemotherapy following conservative surgery is not well understood, approximately one-third of patients with stage I MT-MCT received adjuvant chemotherapy following FSS. To maximize fertility preservation, conservative surgery followed by adjuvant chemotherapy may be a reasonable choice. However, we should keep in mind the possibility of the publication bias. Namely, it is unlikely that any surgeon would have report a case in which the patient have recurred and/or died from disease. It is possible that patients who have favorable clinical outcome tends to be reported particularly in a case study.

The limitations of the current report are those associated with any retrospective study, containing a possibility of selection bias, and treatment heterogeneity. Indeed, because our cases were accumulated from multiple institutions over a long time, the treatment protocol for MT-MCT, including the salvage chemotherapy, was not necessarily consistent. Moreover, the most weakness of our current study was based on small-scale patient number due to the fact that experience of patients with MT-MCT was extremely rare. Particularly, in multivariable analysis, including a variety of clinicopathologic indicators, the significance of the residual tumor disappeared as an independent prognostic factor (HR: 0.76, P= 0.67, 95% CI: 0.21-2.74). Probably, because the stage and residual tumor were strongly correlated with each other, we speculate that the “multicolinearity” might be observed. We also think that it might be related to the small-scale sample number as type II error. In this context, this investigation is a hypothesis-generating study on the application and expectable effect of FSS in young women with MT-MCT. We would like to reevaluate the results of the current examination in other future study, accumulating more and more patients with MT-MCT.

In conclusion, although the criteria for FSS for early stage MT-MCT is not well established, FSS may be an alternative to radical surgery for women of child-bearing age with early-stage MT-MCT at childbearing age. We only report that the prognosis of patients with early-stage MT-MCT who underwent FSS may be not inferior to that of patients who received radical surgery. Accordingly, FSS for women with MT-MCT should be carefully selected based on comprehensive informed consent of the potential increased risk of recurrence. Further evidences regarding MT-MCT is required to safely suggest FSS for patients who wish to preserve fertility.

## PATIENTS AND METHODS

Between January 1986 and December 2016, patients with MT-MCT were accumulated by the Tokai Ovarian Tumor Study Group (TOTSG), consisting of Nagoya University Hospital and 13 affiliated institutions cooperating under the central pathological review system. Patients with EOC, malignant germ cell tumors or borderline epithelial tumors were excluded.

We collected and analyzed data from the medical records and clinical follow-up visits, including age, FIGO stage (International Federation of Gynecology and Obstetrics: 1988), type of surgery, tumor histology, tumor marker, adjuvant chemotherapy, date of recurrence, date of operation, date of death or last follow-up and pregnancy outcome. Two patients were excluded from this analysis due to insufficient information regarding surgical procedure and lost to follow-up immediately after operation. Histological slides were reexamined by our central pathological review system with no knowledge of the patients’ data.

The surgical management plan for patients was determined by staging at initial surgery. In principle, complete radical surgery including total hysterectomy and bilateral salpingo-oophorectomy with peritoneal staging and lymphadenectomy was performed except for in patients who wished to preserve fertility at the age of <45 years old or had difficulty in undergoing radical surgery due to complications. In this study, we defined both complete radical surgery as mentioned above and incomplete surgery including total hysterectomy and bilateral salpingo-oophorectomy, as radical surgery. FSS was fundamentally defined as unilateral oophorectomy or cystectomy with or without omentectomy, together with cytology of ascites and evaluation of any suspected metastasis, including lymph nodes and intraperitoneal dissemination by preoperative or postoperative CT scan.

In order to evaluate oncological outcome of early stage MT-MCT, patients with stage I-II MT-MCT were divided into three groups: group A, patients who underwent FSS with eligible criteria as shown below; group B, patients who were less than 45 years old and underwent radical surgery including total hysterectomy and bilateral salpingo-oophorectomy; group C, patients who were more than 46 years old at the initial surgery. Eligible patients for group A were: (1) under 45 years old at the time of the initial surgery, (2) those who strongly desired to retain fertility, (3) were informed of the pros and cons of FSS, and signed a consent form. Following initial treatment, all patients underwent a periodic follow-up, including an ultrasonographic scan and CT scan. The overall survival (OS) was determined by the duration between the date of initial surgery and the last date of follow-up or death due to any reason. Disease-free survival (DFS) was defined as the duration between the date of surgery and that of recurrence or the last follow-up. The clinicopathological features among groups were evaluated by Chi-square tests. Survival curves were drawn using the Kaplan-Meier method and compared with the Log-rank test. Univariate and multivariate analyses were performed with Cox's proportional hazard model. A P-value of <0.05 was defined as significant.

## Abbreviations

MT-MCT: malignant transformation arising from mature cystic teratoma of the ovary
FSS: fertility-sparing surgery
OS: overall survival
DFS: disease-free survival
EOC: epithelial ovarian cancer
